# What is Needed to Improve Food Sales in Schools? Food Vendors’ Opinion from El Salvador

**DOI:** 10.3389/fpubh.2015.00168

**Published:** 2015-07-01

**Authors:** Caroline Hilari, Margarita Franco

**Affiliations:** ^1^School Health and Nutrition Unit, Department of Child Education and Protection, Save the Children Federation Inc., La Paz, Bolivia; ^2^Save the Children International, El Salvador Country Office, San Salvador, El Salvador

**Keywords:** school nutrition, sale regulations, child obesity, food vendors, save the children

Save the Children International (SCI) implements a school health and nutrition program in 45 schools in El Salvador. The program’s overall goal is to increase the consumption of protein and micronutrients while reducing intake of sugar and fats among school children.

Save the Children International started this program because the Latin America and Caribbean region is at the forefront of the double burden of malnourishment, with rocketing rates of overweight, obesity, and high rates of stunting at the same time (1). Latin Americans tend to decrease healthier foods and consume more fat and sugar when emerging from poverty (2). In the 30-year span from 1990 to 2020, Central America is projected to double its prevalence of overweight and obesity among children under five (3). School children are at a crucial age for setting eating and physical activity habits, which in turn determine their nutritional status and long term health, education, and economic productivity. Overweight and obesity have become a public health problem in El Salvador. According to a recent study in 2014 (4), 30% of 389 young adolescents (7th–11th graders) were overweight and within that group, 8% were obese, comparing to only 2% of children being underweight or very thin. In a previous study in 2012, 23% of 1st and 2nd graders were reported to be overweight or obese (5).

El Salvador has a governmental recommendation regulating the sale of soft drink in schools (5, 6), which has been proposed to become a full law (7, 8). Schools stores also have a written contract, which regulates the vendor’s services, based on a model contract from the Ministry of Education.

Save the Children International’s school store intervention aims to improve the quality of food that children *buy* at school. SCI trains and supports student brigades to enforce the current food sale regulation. Thus, monitoring and control lies in the hands of students, which fosters child participation. SCI’s program is specifically known for active child participation in design, implementation, and monitoring (9). In addition, there is great emphasis on food hygiene and safe handling, through training of the food vendors (10). The indicator of success is the proportion of schools with stores selling fruit. Of the 45 schools, we found 38 stores selling fruit in 2012 and 41 in 2013. We were intrigued to learn more about the determinants for healthy food stores on the ground. Therefore, SCI conducted key informant interviews in 2014, in six SCI supported schools, three in the Izalco municipality (Sonsonate Department), and the other three in San Pedro (La Paz Department). In each municipality, we chose one urban and two rural school food vendors. We asked open-ended questions in three thematic areas of interest:

(1) Products most sold and products with greatest profit.
(2) Training content and usefulness.
(3) Knowledge and opinion about sales regulation and enforcement.

Visits were preannounced to the school director to make sure that schools were open and key informants available, but not to the school vendor. We also observed the type of food on sale during these interviews. Vendors gave informed verbal consent to being interviewed. Confidentiality was ensured through conducting the interview in a private setting, where directors or parents could not overhear the conversation.

All six schools visited had fruit and self-made juice (fresh fruit, water, and sugar) on sale. However, two stores sold also soft drinks permanently and two others said they sold soft drinks once a week or occasionally. Those who were selling soft drinks permanently also had unhealthy or “junk” foods such as industrially produced chips (“churros” in Central American Spanish). On the other hand, the stores who were NOT selling soft drinks were selling homemade foods such as sandwiches, cooked or baked traditional foods. The following describes the key motivators and barriers for compliance with the national policy, according to food vendors.

## Finding a Profitable Healthy Food

In this context, homemade fruit juice and fresh fruit apparently give greatest profit margin, mentioned by four vendors. Especially if they take advantage of availability and low prices during harvest time, they can maximize their profit and it is higher than for chips and soft drinks.

I have a little field, my mom collects the mango, so I don’t spend basically anything (on fruit), it is all for the profit. (Divina Providencia)

Fruit juice and fresh fruit gives most profit. Economically, you don’t earn anything out of candy, not a lot of profit. (Las Isletas)

On the other side, fruit preparation is more work for the vendor, in some cases required hiring additional helpers.

For the costs, it’s good for me. But fruit is more work. You have to get up really early to get (harvest or buy) the fruit. That said, you have to work for the fruit. (Divina Providencia)

## Children Staying Inside the School Premises

Vendors expressed great concern about children being allowed to go out during school hours or the lack of a fence around school premises. They said that this usually leads to buying junk food in nearby stores on the street and then bring it into school premises. This frustrates the in-school vendors because they lose profit and it counteracts the regulation.

This school doesn’t have a wall, and outside there is everything (all type of food products). I am paying my rent in here, if I stop selling this (junk food), the vendor outside of the school earns more. They would have to put a wall around the school so that kids don’t go out. (San Marcelino)

The kids just go out and buy chips, the door is open, they buy it from the sale posts in the street, anything they want. But as it is not allowed, I don’t sell (chips). (Talcomunca)

They buy the chips from outside (of the school), they buy it on their way. (Izalco)

Children leaving school has other implications too in the Salvadorian context: there is a danger of road accidents and there is great danger of criminal violence, including recruitment into armed groups. This was mentioned by several principals and parents.

## No Aggressive Marketing Strategies by Soft Drink Producers

In one school, Pepsi Cola was counteracting the national policy, by giving support with sport equipment, benches, tables, and other furniture. These supplies are highly valued and would be lost if a “no soft drink” policy were enforced.

They were forbidding the soft drinks, but it is impossible. Some providers, like Pepsi Cola, support our school centers, they support our schools stores, they give footballs, furniture. You can’t forbid them to sell their product, right? (School director, Las Isletas)

## Continuous Control of the School Store

In one very healthy food store, the school principal had a strong and clear authority, with a clear commitment to healthy food on school premises:

The director here is quite strong, some parents have complained. We even cannot sell the same stuff every day; here we have to say what she tells us, she gives us the menu. The director is the one who reigns (here). (Sega)

The other important feature is the contract with the parent association:

I like things to be correct, I like complying. If everything is the same as before (selling chips and soft drinks), then we are not complying, right? Legal is legal. They should give us the contract at the beginning of the year, so that we know the conditions. (Divina Providencia)

## More Knowledge about Junk Food

Knowledge, modified by personal experience, influences the vendors’ attitudes and motivation to limit the sale of junk food. All vendors knew that soft drinks are “forbidden” to sell. Above that, some vendors were conscious of the addictive properties of artificially flavored chips and soft drinks, especially Coca Cola. The main harmful content of junk food is considered to be high content in fat, salt, artificial flavors, and colors. Vendors cite hyperactivity as a main health problem due to junk food consumption:

Chips makes something explode inside of the children, they become hyperactive. Kids want chips because it is addictive, my son was addicted to soft drinks, but I took it away because I saw a program on TV and then I took it away from him. I agree with not selling junk food, because it is really bad for everything, the soft drinks are harmful for the bones. (Izalco)

On the other hand, there is very little consciousness among vendors about the role of sugar in overweight, obesity, and dental caries. Only one vendor mentioned this effect:

The ingredients do harm, they make the sugar go up in the blood. At nine years of age they already have (high) blood sugar, hypertension. Super fat, but not at all well-nourished. (Divina Providencia)

During observation, particularly in those stores which did not sell soft drinks and junk food, we saw more homemade foods on sale such as juices or sweets. The very high sugar content points to the fact that homemade products are not necessarily healthier than industrially produced foods. Additionally, food vendors need to be informed about the harmful consequences of high sugar content in children’s diet.

Finally, most vendors had received some training on food hygiene by the public health service, supported by SCI. However, only one of the vendors remembered a message about food quality from the training course:

They taught us the importance of selling natural foods. (Divina Providencia)

## Parent and Children’s Attitude to Healthy Food

Several vendors mentioned that children would be less likely to bring junk food to school if taught and motivated by an active student volunteer brigade. They also mentioned parent control in the home, so that buying food becomes more conscious about health impacts.

The student brigade comes, they check that all the food is covered. (San Marcelino)

There is a Health and Nutrition committee. The parent association should be motivating the children about what is harmful to them. (Las Isletas)

Two years ago, we had a very active parent association and student brigade. The Save the Children technician had real leadership, he motivated the kids. Every week the brigade would control us. The kids couldn’t go out to buy in the street. “You have your store here, you don’t have any reason to go out to the street” they would tell them. And they also had festivals to motivate kids for good nutrition. We were famous for having a healthy school store; I could even sell homemade sweets. (Divina Providencia)

We propose to classify the “ecologic” or enabling environmental conditions for healthy food in schools into three levels of action:

**Table:** 

Level of action	Enabling condition for healthy food
Vendors	Finding a profitable healthy food
School administration	Keeping children inside the school premises
	Controlling or enforcing the healthy food regulation
	Keeping soft drink marketing out of schools
Civil society, including NGOs	Vendors’ knowledge about healthy food
	Children’s and parents’ attitude toward healthy food

We had initially used “selling fruit” per observation as the main indicator for clarifying schools stores as compliant with regulations. However, after analysis of the interviews and observations, we propose to use “not selling soft drinks” as indicator, because the absence of soft drinks was a fairly good differentiator variable for a healthy food store classification.

In the future, we propose to expand our school store program intervention so that vendors have a facilitating environment to be able to comply with the national regulation.

## Author Contributions

CH provided the initial framework, conducted, translated, and analyzed the interviews. MF coordinated data collection in the field, monitoring, and program interventions. Both worked on the final version of this article. We wish to thank Mario Alvarado, Ma-luschka Colindres, Ludin de Chávez, Mara Ruiz, and several drivers for effective collaboration.

## Conflict of Interest Statement

This research was conducted in the absence of any commercial or financial relationships that could be construed as a potential conflict of interest. We did not at any time receive payment or services from a third party for any aspect of the submitted work.
